# Utility of Repeating Blood Cultures in Candidemia: Insights From 2 Large Clinical Data Repositories

**DOI:** 10.1093/cid/ciag216

**Published:** 2026-05-14

**Authors:** Hisato Yoshida, Max W Adelman, Francis Ifiora, María Alejandra Pérez, Marilyn Niravath, Hitoshi Yoshimura, Stephen L Jones, Cesar A Arias, Masayuki Nigo

**Affiliations:** Center for Infectious Diseases, Houston Methodist Research Institute, Houston, Texas, USA; Department of Dentistry and Oral Surgery, Unit of Sensory and Locomotor Medicine, Division of Medicine, Faculty of Medical Sciences, University of Fukui, Fukui, Japan; Center for Infectious Diseases, Houston Methodist Research Institute, Houston, Texas, USA; Division of Infectious Diseases, Houston Methodist Hospital, Houston, Texas, USA; Department of Medicine, Weill Cornell Medical College, NewYork, New York, USA; Division of Pulmonary, Critical Care, and Sleep Medicine, Department of Medicine, Houston Methodist Hospital, Houston, Texas, USA; Center for Infectious Diseases, Houston Methodist Research Institute, Houston, Texas, USA; Center for Infectious Diseases, Houston Methodist Research Institute, Houston, Texas, USA; Center for Health Data Science and Analytics, Houston Methodist Hospital, Houston, Texas, USA; Department of Surgery, Weill Cornell Medical College, NewYork, New York, USA; Department of Dentistry and Oral Surgery, Unit of Sensory and Locomotor Medicine, Division of Medicine, Faculty of Medical Sciences, University of Fukui, Fukui, Japan; Center for Health Data Science and Analytics, Houston Methodist Hospital, Houston, Texas, USA; Department of Surgery, Weill Cornell Medical College, NewYork, New York, USA; Center for Infectious Diseases, Houston Methodist Research Institute, Houston, Texas, USA; Division of Infectious Diseases, Houston Methodist Hospital, Houston, Texas, USA; Department of Medicine, Weill Cornell Medical College, NewYork, New York, USA; Center for Infectious Diseases, Houston Methodist Research Institute, Houston, Texas, USA; Division of Infectious Diseases, Houston Methodist Hospital, Houston, Texas, USA; Department of Medicine, Weill Cornell Medical College, NewYork, New York, USA; Department of Infectious Diseases, Faculty of Medical Sciences, University of Fukui, Fukui, Japan

**Keywords:** candidemia, blood culture

## Abstract

We evaluated utility of repeating blood culture for candidemia using 2 electronic health record datasets. Among 1386 candidemia episodes, repeated blood culture detected 23% of episodes at Houston Methodist Hospital and 45% in Medical Information Mart for Intensive Care IV after the initial negative cultures. These findings support repeating blood cultures when candidemia is highly suspected.

Candidemia is one of the most common and life-threatening bloodstream infections in hospitalized and critically ill patients, with mortality rates as high as 40% [1]. Blood cultures remain the standard diagnostic method for detecting candidemia. However, the sensitivity of blood cultures is limited, and they detect only about half of invasive candidiasis, such as deep-seated candidal infections [2, 3].

In certain bacterial infections (eg infective endocarditis), repeat blood cultures may be considered to increase the sensitivity of identifying the causative organism [4]. Given the limited sensitivity of blood cultures for candidemia, we hypothesized that repeating blood cultures in patients with suspicion of bloodstream infection due to *Candida* spp. may increase detection of these organisms. Using 2 large patient databases, we aimed to estimate the incidence of candidemia detected by repeat blood cultures within 7 days following an initial negative blood culture.

## METHODS

### Study Design and Study Population

We conducted a retrospective cohort study utilizing 2 electronic health record (EHR) databases: the Houston Methodist Infectious Diseases EHR repository from June 2016 until June 2023 and the Medical Information Mart for Intensive Care IV (MIMIC-IV) version 3.1 [5] (Supplementary Material). All patients who underwent blood culture testing were extracted from each dataset. For each patient, the first blood culture collection was designated as the index time (day 0). Candidemia was defined as any blood culture yielding *Candida* spp. within 7 days after the index time (Supplementary Figure 1). Because multiple blood cultures may be obtained on the same date for a given patient, all cultures collected on the same date were treated as a single blood culture episode, with the earliest collection time retained as the representative episode. To further evaluate the utility of repeat blood cultures within 24 hours, we conducted a more granular analysis of the timing of subsequent blood cultures during the period. If any culture obtained on that date yielded *Candida* spp., the patient-day was classified as *Candida*-positive. Any blood culture obtained outside this 7-day window triggered assignment of a new index time and establishment of an additional 7-day observation window as a new event. We used a 7-day episode window to define a candidemia episode, grouping repeated blood cultures within this interval as a single outcome episode. Although the Centers for Disease Control and Prevention/National Healthcare Safety Network (CDC/NHSN) uses a 14-day repeat infection timeframe, a 7-day window was used in this study, as a longer window may count the newly acquired invasive candidal infections after day 0 [6]. To characterize patients’ clinical backgrounds, we additionally extracted clinical events occurring before the index time, including laboratory results, medication administrations, and hospitalization information (including intensive care unit [ICU] admissions). Abnormal vital signs suggesting infections at the time of the index blood culture were assessed only in the Houston Methodist Hospital System (HMHS) dataset, as MIMIC-IV does not capture vital signs outside the ICU. These signs included fever (≥100.4°F), hypothermia (≤95.0°F), and receipt of vasopressor support within 48 hours before the index culture. Candida colonization was defined as the identification of *Candida* spp. or yeast without identification of species from nonsterile cultures. Patients who received any systemic antifungal agent, such as prophylaxis, within 24 hours prior to the index time were excluded from the analysis.

### Outcomes

The primary outcome was candidemia, defined as detection of *Candida* spp. in a blood culture within 7 days of each index blood culture collection. To characterize the temporal pattern of candidemia detection, we constructed Kaplan–Meier (KM) curves. For each index time, the event was defined as the date of the first *Candida*-positive blood culture, and the time to event (days) was used in KM analysis.

### Ethics

The study protocol was approved by the Houston Methodist Institutional Review Board (Project number: PRO00037862).

## RESULTS

During the study period, a total of 761/211 006 (0.36%) and 625/107 263 (0.58%) candidemia events were identified from blood cultures in HMHS and MIMIC-IV datasets, respectively. Fungal blood cultures were utilized in only 2.1% (60/2834) in the HMHS cohort and 2.4% (61/2534) in the MIMIC-IV cohort (Supplementary Table 1). Supplementary Table 2 summarizes the clinical characteristics of patients in the HMHS and MIMIC-IV cohorts. We further analyzed the number and timing of blood culture until candidemia diagnosis. Substantial variability in the timing of repeat cultures was observed (Supplementary Figures 2–4). Among candidemia patients diagnosed within 24 hours, repeat blood cultures within the same 24-hour period identified 35 patients (4.6%) in the HMHS cohort and 59 patients (9.4%) in the MIMIC-IV cohort. Notably, in HMHS, 122 patients (18.6%) with candidemia identified within 24-hour period did not have any abnormal vital signs at the time of blood cultures.

**Figure 1.**
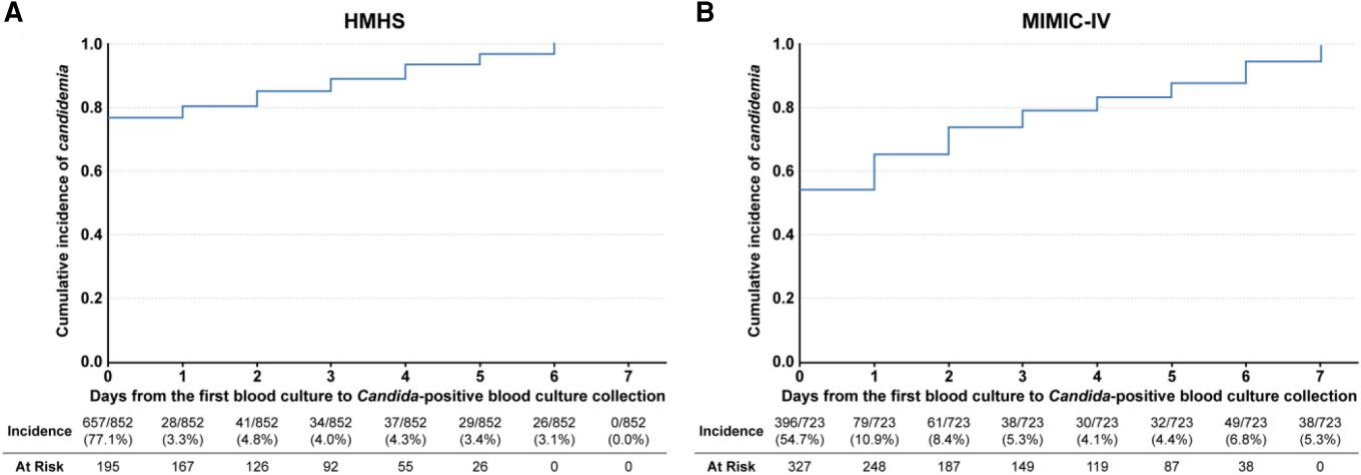
Cumulative incidence of candidemia following the first blood culture in hospitalized patients. *A*, Houston Methodist Hospital System (HMHS) cohort and *B*, Medical Information Mart for Intensive Care IV (MIMIC-IV) cohort. The plots show the cumulative incidence of candidemia among patients who developed candidemia after their first blood culture, stratified by cohort. The *x*-axis represents the number of days from the first blood culture to the first *Candida*-positive blood culture collection. The risk table reports the interval-specific number of new *Candida*-positive blood cultures (Incidence) and the number of patients still event-free and under observation at each time point (At risk).

As shown in Figure 1, a substantial number of candidemia cases were identified by repeat blood cultures (195/852 [22.9%] and 327/723 [45.2%] in HMHS and MIMIC-IV, respectively) after the initial day. This indicates that nearly half of candidemia cases in MIMIC-IV—and a notable proportion in HMHS—were potentially missed by the initial blood culture. Supplementary Table 3 summarizes bacteremia cases concomitant or preceding to candidemia. In both cohorts, preceding bacteremia was uncommon (5.9% [50/852] in HMHS, 12.7% [92/723] in MIMIC-IV), and excluding these cases did not significantly change the proportion of candidemia diagnosed by repeat blood cultures (HMHS: 18.1% [145/802]; MIMIC-IV: 39.6% [250/631]).

As shown in Supplementary Figure 5, similar patterns were observed in both ICU and non-ICU settings. In HMHS, repeat cultures identified ∼23% of cases in both settings, while in MIMIC-IV, about 45% of ICU and non-ICU cases were detected only on subsequent cultures within 7 days.

## DISCUSSION

In this study, using 2 large EHR databases, we found that a substantial proportion of candidemia cases were identified through repeat blood cultures. This pattern was consistent in both ICU and non-ICU settings: nearly half of cases in the MIMIC-IV cohort and approximately one-quarter of cases in the HMHS cohort were diagnosed with subsequent blood cultures. These findings highlight the limited sensitivity of blood cultures and underscore the potential diagnostic value of repeating blood cultures when clinical suspicion persists. We did not observe a consistent temporal pattern in the timing of repeat cultures, which precluded identification of an optimal interval for repeat blood culture collection.

In this study, 22.9% of candidemia events in the HMHS cohort and 45.2% in the MIMIC-IV cohort were detected only through repeat blood cultures. This finding is significantly different from prior studies of bacterial bloodstream infections. In a large analysis of more than 23 000 blood culture bottles, only 2.6% of cases were identified on repeat cultures after excluding contaminants and outside of endovascular infections [7]. Another study reported that only 5 out of 176 patients (2.8%) who had blood cultures in the emergency room had subsequent positive culture after the negative initial blood cultures, most commonly involving *Enterococcus* species [8]. These differences are likely driven by better sensitivity of blood cultures on bacteremia, and empirical antibacterial therapy is often given after the initial blood cultures, compared to empirical antifungal therapy.

A substantial proportion of candidemia patients was identified outside the ICU. Specifically, 69.1% of such initial cultures in the HMHS cohort and 67.6% in the MIMIC-IV cohort were collected in non-ICU settings. Although several scoring systems have been developed to predict candidemia, most have focused primarily on ICU populations [9–11]. Given that many candidemia cases occur outside the ICU and are detected on subsequent blood cultures, there is a critical need for prediction models that span all inpatient wards and can predict candidemia even following initially negative cultures. Early detection is essential, as these cases carry significant mortality risks.

This study has several limitations. First, its retrospective design may introduce unmeasured confounding as well as variability in blood culture collection practices. Furthermore, patients who underwent repeat blood cultures may have represented a higher-risk population, introducing potential selection bias. Considering the 7-day diagnostic window, patients may develop candidemia after initial negative blood cultures. However, only small number of candidemia patients had documented bacteremia preceding to candidemia. Second, we were unable to include patients highly suspected of candidemia without positive blood cultures. Although β-D-glucan can support the diagnosis of invasive candidal infections, its limited specificity precluded its use as an outcome measure. Only a limited number of patients were tested for serum (1→3)-β-D-glucan in HMHS datasets, and the test was not available in MIMIC-IV. Rapid diagnostics, such as direct blood molecular tests [12], were not available in our datasets. Lastly, although our findings highlight the importance of repeat blood cultures in patients with suspected candidemia, this strategy may lead to unnecessary testing given the overall rarity of candidemia. Improved risk-stratification tools are needed to guide both empirical antifungal therapy and the judicious use of repeat blood cultures.

## CONCLUSIONS

A substantial proportion of candidemia cases were identified through repeat blood cultures, highlighting their diagnostic yield in real-world practice while acknowledging the limitations of retrospective data. Repeat cultures should be considered when concern for candidemia persists. Because many cases occurred outside the ICU, future risk-stratification models should encompass all inpatient wards. Further studies are warranted to confirm the findings.

## Supplementary Material

ciag216_Supplementary_Data

## Supplementary Data

Supplementary materials are available at *Clinical Infectious Diseases* online. Consisting of data provided by the authors to benefit the reader, the posted materials are not copyedited and are the sole responsibility of the authors, so questions or comments should be addressed to the corresponding author.
