# Supplementary material for: Utility of Repeating Blood Cultures in Candidemia: Insights From 2 Large Clinical Data Repositories
Source: Clin Infect Dis. 2026 May 14;83(1):e105–8. doi: 10.1093/cid/ciag216 (PMC13393118; doi:10.1093/cid/ciag216)
Supplement: ciag216_Supplementary_Data [file ciag216_supplementary_data.docx]

**Supplementary Information**

**Database utilized in this study**

**Supplementary Figure 1:** **Schematic Explanation of a 7-day Candidemia Episode**

**Supplementary Table 1 Distribution of *Candida*-positive blood culture sets obtained concurrently with routine and fungal cultures, defined within a 3-hour window**

**Supplementary Table 2:** **Clinical characteristics of patients with candidemia**

**Supplementary Figure 2: Distribution of blood culture set counts until Candida-positive blood culture in HMHS and MIMIC-IV**

**Supplementary Figure 3: Timing of repeated blood culture sets in patients with candidemia (HMHS)**

**Supplementary Figure 4: Timing of repeated blood culture sets in patients with candidemia (MIMIC-IV)**

**Supplementary Table 3: Bacterial species detected in blood cultures within 7 days prior to Candida-positive blood culture**

**Supplementary Figure 5:** **Cumulative incidence of candidemia by ICU status in two cohorts**

**Database utilized in this study**

**Houston Methodist Infectious Diseases Electronic Health Record Repository (HM-IDeHR)**

Houston Methodist Hospital System comprises a total of eight acute care hospitals in the greater Houston Area [1]. HM-IDeHR is a regularly updated longitudinal EHR database, which was specifically developed to evaluate infectious diseases-related outcomes.

**Medical Information Mart for Intensive Care IV (MIMIC-IV) version 3.1**[2]

MIMIC-IV is a large, publicly accessible critical care database derived from patient records at the Beth Israel Deaconess Medical Center in Boston, USA.

**Supplementary Figure 1:** **Schematic Explanation of a 7-day Candidemia Episode**


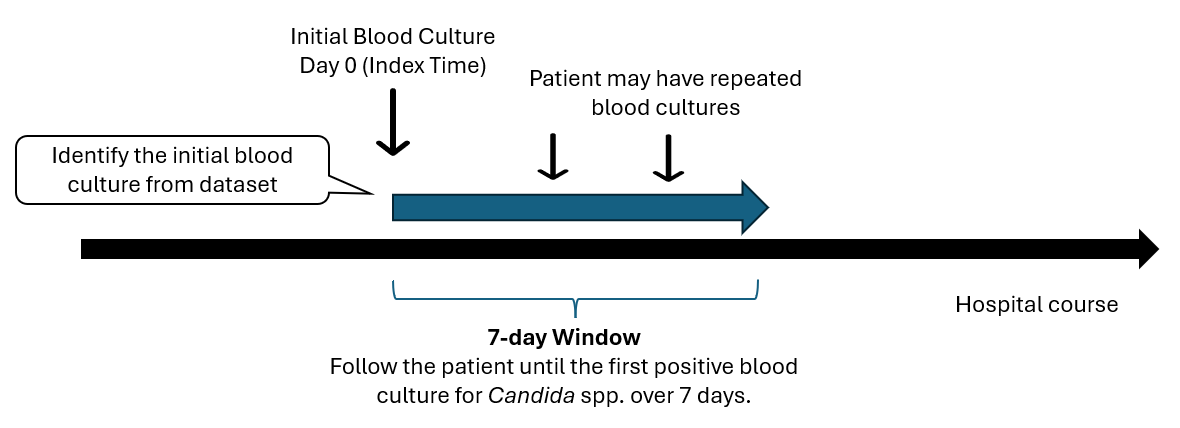
Using electronic health record datasets, we identified the first blood culture obtained for each patient and defined this as day 0 (index time). For patients whose initial blood cultures were negative or positive for non-Candida pathogens, we followed them for up to 7 days to capture subsequent blood cultures. The outcome of interest was the detection of Candida spp. from either the initial or any repeat blood culture within this 7-day follow-up period. Patients could contribute multiple, non-overlapping 7-day event windows if they experienced more than one episode of candidemia during the study period.

**Supplementary Table 1. Distribution of *Candida*-positive blood culture sets obtained concurrently with routine and fungal cultures, defined within a 3-hour window**

| *Candida*-positive blood culture sets | HMHS (N=24) | MIMIC-IV (N=18) |
| --- | --- | --- |
| Routine blood culture (+), Fungal blood culture (-) | 16 (77.1) | 4 (22.2) |
| Routine blood culture (-), Fungal blood culture (+) | 8 (33.3) | 1 (5.6) |
| Routine blood culture (+), Fungal blood culture (+) | 0 (0) | 13 (72.2) |

**Abbreviations:** HMHS, Houston Methodist Hospital System; MIMIC-IV, Medical Information Mart for Intensive Care IV

This table summarizes *Candida*-positive blood culture sets in which both routine and fungal blood cultures were obtained concurrently, defined as specimens collected within the same 3-hour window. Routine culture–negative but fungal culture–positive cases accounted for 33% (8/24) in the HMHS cohort and 6% (1/18) in the MIMIC-IV cohort.

**Supplementary Table 2. Clinical characteristics of patients with candidemia**

|  | **HMHS** | **MIMIC-IV** |
| --- | --- | --- |
|  | Candidemia group N=761 | Candidemia group N=625 |
|  | N (%) | N (%) |
| **Age** |  |  |
| 18-24 | 6 (0.8) | 8 (1.3) |
| 25-34 | 34 (4.5) | 46 (7.4) |
| 35-44 | 52 (6.8) | 64 (10.2) |
| 45-54 | 83 (10.9) | 92 (14.7) |
| 55-64 | 191 (25.1) | 106 (17.0) |
| 65-74 | 207 (27.2) | 153 (24.5) |
| 75-84 | 141 (18.5) | 115 (18.4) |
| 85-94 | 42 (5.5) | 41 (6.5) |
| 95-104 | 1 (0.1) | 0 (0.0) |
| **Gender** |  |  |
| Male | 404 (53.1) | 342 (54.7) |
| **Ethnicity** |  |  |
| Hispanic | 125 (16.4) | 22 (3.5) |
| Non-Hispanic | 623 (81.9) | 603 (96.5) |
| **Race** |  |  |
| White | 467 (61.4) | 412 (65.9) |
| African American | 215 (28.3) | 79 (12.6) |
| Asian | 40 (5.3) | 11 (1.8) |
| **Selected comorbidities^1^** |  |  |
| Cerebral vascular diseases | 115 (13.5) | 53 (8.5) |
| Congestive heart failure | 145 (17.0) | 111 (17.8) |
| Chronic pulmonary diseases | 214 (25.1) | 160 (25.6) |
| Cirrhosis | 61 (7.2) | 41 (6.6) |
| Chronic kidney disease | 258 (30.3) | 130 (20.8) |
| Hypertension | 315 (37.0) | 263 (42.1) |
| Diabetes mellitus | 236 (27.7) | 177 (28.3) |
| Malignancy | 209 (24.6) | 132 (21.1) |
| Hematologic malignancy | 24 (3.1) | 37 (5.9) |
| Leukocytosis |  |  |
| 10,000< (/µL) | 387 (50.9) | 234 (37.4) |
| 11,000< (/µL) | 351 (46.1) | 208 (33.2) |
| Neutropenia |  |  |
| ≤500 (/µL) | 2(0.3) | 21 (3.4) |
| 500-1000 (/µL) | 4 (0.5) | 9 (1.4) |
| 1000-1500 (/µL) | 8 (1.1) | 9 (1.4) |
| HIV/AIDS | 8 (0.9) | 6 (1.0) |
| Solid organ transplantation | 35 (4.6) | 11 (1.8) |
| Hematopoietic stem cell transplantation | 1 (0.1) | 7 (1.1) |
| **Fever (>= 100.4F/38.0˚C)** | 375 (49.2) | -^2^ |
| **Temperature ≥ 98.5F/37.5˚C** | 581 (68.2) | -^2^ |
| **Hypothermia ≤ 95.0F/35˚C** | 36 (4.2) | -^2^ |
| **Central venous catheter use^3^** | 98 (12.9) | 124 (19.8) |
| **Total parenteral nutrition** | 152 (20.0) | 83 (13.3) |
| **Hemodialysis** | 14 (1.8) | 44 (7.0) |
| **Prescription of medicine** |  |  |
| Broad-spectrum antibiotics^4^ | 175 (23.0) | 302 (48.3) |
| Corticosteroids^5^ | 320 (42.1) | 146 (23.4) |
| Immunosuppressants^6^ | 40 (5.3) | 32 (5.1) |
| **Candida colonization^7^** | 5 (0.7) | 125 (20.0) |
| **Candida species detected in blood cultures (Top5)** |  |  |
| *Candida albicans* | 273 (35.9) | 264 (42.2) |
| *Nakaseomyces glabrata* ^8^ | 231 (30.3) | 195 (31.2) |
| *Candida parapsilosis* | 110 (14.4) | 85 (13.6) |
| *Candida tropicalis* | 73 (9.6) | 44 (7.0) |
| *Candida auris* | 18 (2.4) | 1 (0.2) |
| **ICU stay at the time of blood culture** | 241 (31.7) | 202 (32.3) |
| **Infectious diseases diagnosis^9^** |  |  |
| Sepsis | 473 (62.1) | 319 (51.0) |
| Pneumonia | 280 (36.8) | 159 (16.6) |
| Urinary tract infection | 226 (29.7) | 104 (25.4) |
| Abdominal infection | 132 (17.3) | 98 (15.7) |
| Skin soft tissue infection | 70 (9.2) | 55 (8.8) |

**Abbreviations:** HMHS, Houston Methodist Hospital System; MIMIC-IV, Medical Information Mart for Intensive Care IV; ICD-10, International Classification of Diseases, Tenth Revision; LOINC, Logical Observation Identifiers Names and Codes

^1^Selected comorbidities and infectious diseases diagnosis were determined from ICD-10 diagnostic codes. Leukocytosis and neutropenia were calculated using laboratory data identified by LOINC codes.

^2^MIMIC-IV does not have vital signs data in patients outside ICU.

^3^The presence of a central venous catheter was determined from ICD-10 procedure codes.

^4^Broad-spectrum antibiotics were identified based on the Antimicrobial Use and Resistance (AUR) Module published by the U.S. Centers for Disease Control and Prevention (CDC) [3].

^5^Corticosteroids included the following agents: cortisone, hydrocortisone, fludrocortisone, methylprednisolone, prednisone, prednisolone, dexamethasone, and betamethasone.

^6^Immunosuppressants included teriflunomide, pegcetacoplan, ofatumumab, antithymocyte agents, dimethyl fumarate, secukinumab, belumosudil, alemtuzumab, certolizumab, cladribine, pirfenidone, tofacitinib, upadacitinib, tacrolimus, cyclosporine, dupilumab, ustekinumab, benralizumab, tocilizumab, sarilumab, anakinra, methotrexate, azathioprine, lenalidomide, thalidomide, vedolizumab, leflunomide, natalizumab, abatacept, belimumab, mycophenolate, everolimus, baricitinib, mycophenolic acid, sirolimus, temsirolimus, eculizumab, belatacept, adalimumab, etanercept, infliximab, and certolizumab.

^7^Candida colonization included all isolates identified as Candida species or reported as “yeast.”

^8^Candida glabrata and Nakaseomyces glabrata were considered the same species and were collectively reported as Nakaseomyces glabrata.

Supplementary Table 2 summarizes the clinical characteristics of patients with candidemia in the HMHS and MIMIC-IV cohorts. Overall, demographic characteristics and comorbidity profiles were generally similar between the two datasets. Solid organ transplantation was more frequent in the HMHS cohort (4.6% vs. 1.8%), whereas hematopoietic stem cell transplantation and central venous catheter use were more common in the MIMIC-IV cohort (1.1% vs. 0.1% and 19.8% vs. 12.9%, respectively). Broad-spectrum antibiotic exposure was more frequent in MIMIC-IV (48.3%), while corticosteroid exposure was more common in HMHS (42.1%). Candida colonization was rare in HMHS (0.7%) but more frequent in MIMIC-IV (20.0%). Among Candida species detected in blood cultures, Candida albicans was the most common in both cohorts. ICU status at the time of blood culture collection was comparable between cohorts. Sepsis was the most common infectious diagnosis in both datasets, although pneumonia was more frequently observed in HMHS. After including candidemia cases identified within 24-hour period, 87.6% of the cases demonstrated overt signs of infection within 24-hour period in the HMHS dataset. In the MIMIC-IV dataset, a comparable analysis was not performed because vital sign data outside the ICU setting were unavailable.

**Supplementary Figure 2: Distribution of blood culture set counts until Candida-positive blood culture in HMHS and MIMIC-IV**

Panel A shows the distribution in the Houston Methodist Hospital System (HMHS), and Panel B shows the distribution in the MIMIC-IV dataset. The x-axis represents the number of blood culture sets obtained until the *candida*-positive blood culture was identified, and the y-axis represents the number of patients. To facilitate comparison between datasets, the distribution is shown up to four blood culture sets, which captured the majority of candidemia events in both cohorts.


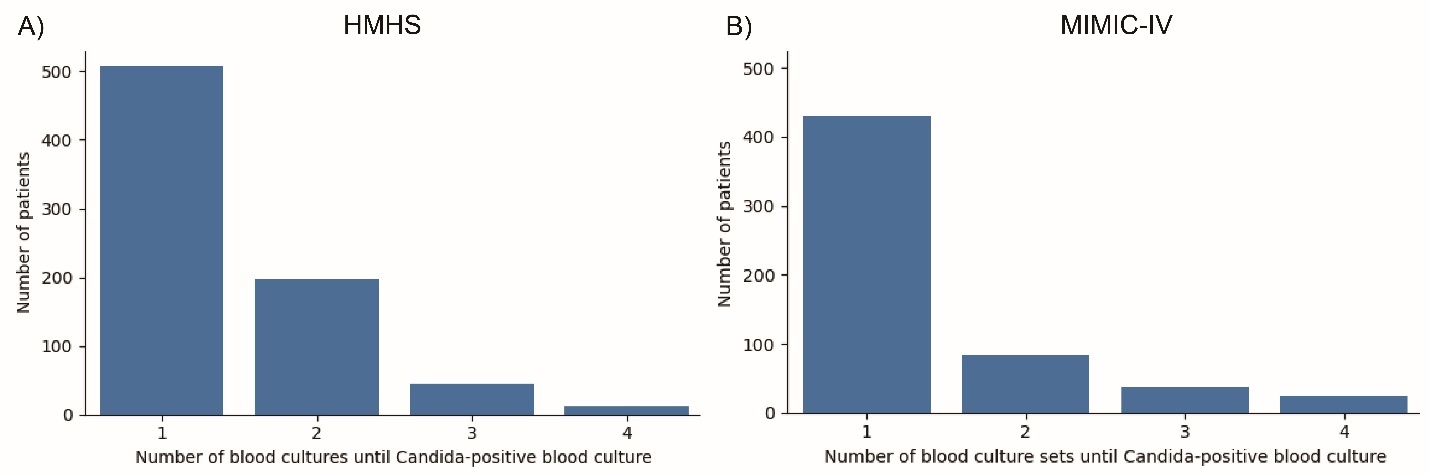


**Supplementary Figure 3: Timing of repeated blood culture sets in patients with candidemia (HMHS)**
Panels A–C show the timing of blood culture sets relative to the index blood culture in patients with exactly 2, 3, and 4 sets, respectively. Blood cultures obtained within 3 hours were considered a single set. The x-axis represents days from the index blood culture, and the y-axis represents the number of blood culture sets. Red bars indicate Candida-positive blood cultures.


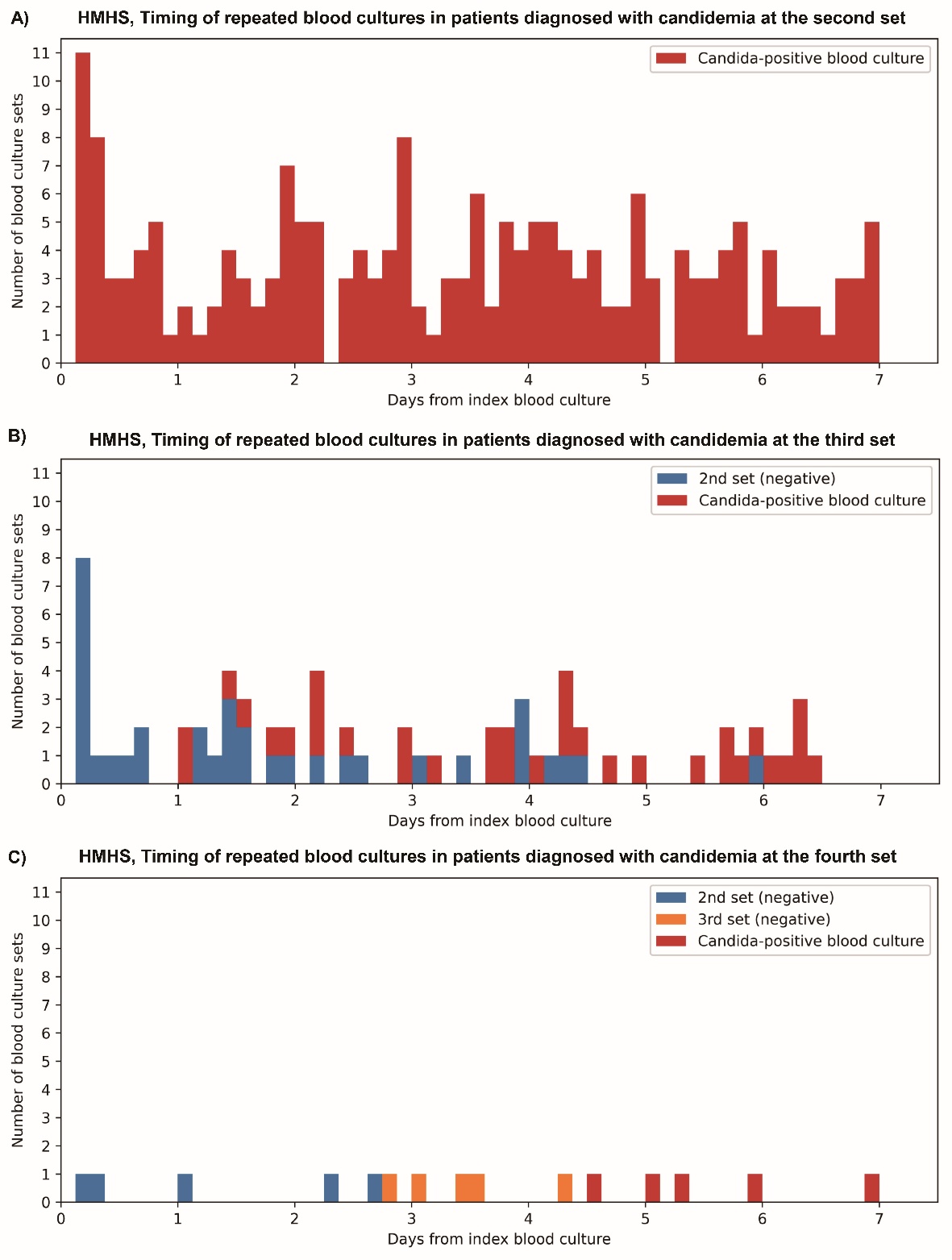


**Supplementary Figure 4: Timing of repeated blood culture sets in patients with candidemia (MIMIC-IV)**
Panels A–C show the timing of blood culture sets relative to the index blood culture in patients with exactly 2, 3, and 4 sets, respectively. Blood cultures obtained within 3 hours were considered a single set. The x-axis represents days from the index blood culture, and the y-axis represents the number of blood culture sets. Red bars indicate Candida-positive blood cultures.


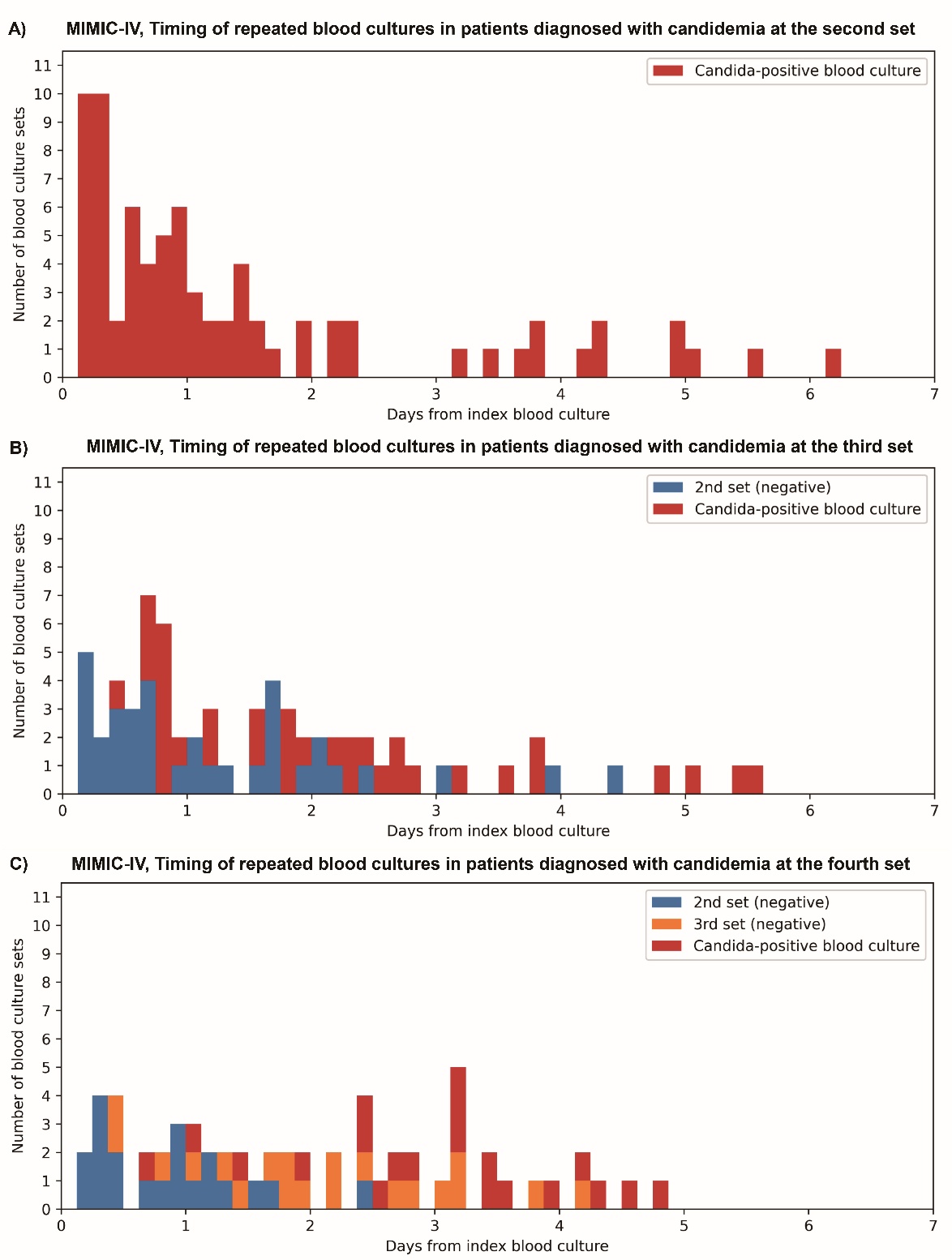


**Supplementary Table 3: Bacterial species detected in blood cultures within 7 days prior to Candida-positive blood culture**

| HMHS (N of episode =852) |  | Days prior to the *candida*-positive blood culture | | | | | | |
| --- | --- | --- | --- | --- | --- | --- | --- | --- |
| Bacteria (Top 5), n (%) | Positive date^1^ | -1 day | -2 days | -3 days | -4 days | -5 days | -6 days | -7 days |
| *Enterococcus* spp | 41 (7.0) | 1 (0.1) | 3 (0.4) | 4 (0.5) | 1 (0.1) | 2 (0.2) | 1 (0.1) | 2 (0.2) |
| *Staphylococcus aureus* | 5 (0.6) | 2 (0.2) | 7 (0.8) | 4 (0.5) | 4 (0.5) | 0 (0.0) | 0 (0.0) | 0 (0.0) |
| *Escherichia coli* | 6 (0.7) | 2 (0.2) | 4 (0.5) | 0 (0.0) | 1 (0.1) | 1 (0.1) | 0 (0.0) | 0 (0.0) |
| *Klebsiella pneumoniae* | 9 (1.1) | 0 (0.0) | 2 (0.2) | 0 (0.0) | 0 (0.0) | 2 (0.2) | 1 (0.1) | 1 (0.1) |
| *Pseudomonas aeruginosa* | 3 (0.4) | 0 (0.0) | 4 (0.5) | 2 (0.2) | 0 (0.0) | 0 (0.0) | 2 (0.2) | 1 (0.0) |
| MIMIC-IV (N of episode =723) |  | Days prior to the *Candida*-positive blood culture | | | | | | |
| Bacteria (Top 5), n (%) | Positive date^1^ | -1 day | -2 days | -3 days | -4 days | -5 days | -6 days | -7 days |
| *Enterococcus* spp | 41 (5.7) | 18 (2.5) | 15 (2.0) | 13 (1.8) | 10 (1.4) | 14 (1.9) | 13 (1.8) | 12 (1.8) |
| *Staphylococcus aureus* | 10 (1.4) | 10 (1.4) | 5 (0.7) | 2 (0.3) | 3 (0.4) | 2 (0.3) | 2 (0.3) | 3 (0.4) |
| *Escherichia coli* | 7 (1.0) | 6 (0.8) | 2 (0.3) | 1 (0.1) | 1 (0.1) | 1 (0.1) | 1 (0.1) | 3 (0.4) |
| *Klebsiella pneumoniae* | 6 (0.8) | 10 (1.4) | 5 (0.6) | 3 (0.4) | 2 (0.3) | 0 (0.0) | 0 (0.0) | 0 (0.0) |
| *Pseudomonas aeruginosa* | 9 (1.2) | 1 (0.1) | 2 (0.3) | 1 (0.1) | 0 (0.0) | 1 (0.1) | 0 (0.0) | 0 (0.0) |

**Abbreviations:** HMHS, Houston Methodist Hospital System; MIMIC-IV, Medical Information Mart for Intensive Care IV

^1^“Positive date” refers to the date of the *candida*-positive blood culture.

This table summarizes the top five bacterial species detected in blood cultures obtained on the day of and within 7 days prior to the *Candida*-positive blood culture in the HMHS and MIMIC-IV cohorts. Values are presented as the number of patients (percentage of the total cohort). Days are shown relative to the *Candida*-positive blood culture date. HMHS (N=852) and MIMIC-IV (N=723) represent the number of candidemia events defined at the event (observation window) level rather than unique patients. Interestingly, *Enterococcus* spp. (6.5-18.8%) were the most common pathogen, followed by *S. aureus* (2.6-5.1%) in both datasets.

**Supplemental Figure 5. Cumulative incidence of candidemia by ICU status in two cohorts**

(A–B) Cumulative incidence of candidemia among ICU patients in the Houston Methodist Hospital Systems (HMHS) cohort (A) and the MIMIC-IV cohort (B).

(C–D) Cumulative incidence of candidemia among non-ICU patients in the HMHS cohort (C) and the MIMIC-IV cohort (D).

The x-axis represents the number of days from the first blood culture to the first Candida-positive blood culture collection.

The risk table reports the interval-specific number of new candida-positive blood cultures (Incidence) and the number of patients still event-free and under observation at each time point (At risk).


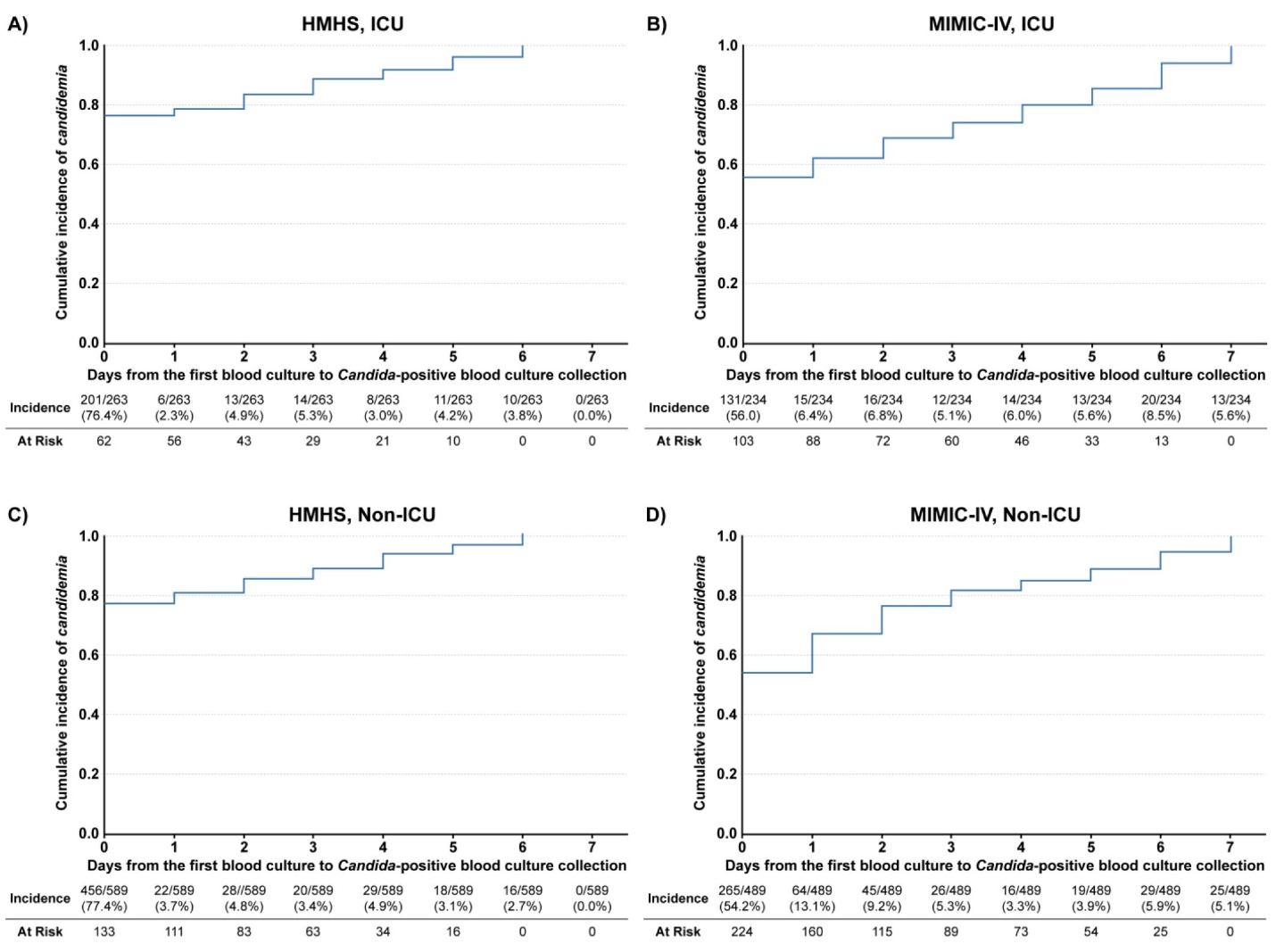


**Reference**

1. Locations | Houston Methodist. Available at: https://www.houstonmethodist.org/locations/. Accessed 18 November 2025.

2. Johnson AEW, Bulgarelli L, Shen L, et al. MIMIC-IV, a freely accessible electronic health record dataset. Sci Data **2023**; 10:1.

3. 2025 NHSN AUR Protocol. Available at: https://www.cdc.gov/nhsn/pdfs/pscmanual/11pscaurcurrent.pdf. Accessed 20 November 2025.
